# What Makes Human Resource Professionals Effective? An Exploratory Lesson From Techno-Based Telco Firms of a Developing Country

**DOI:** 10.3389/fpsyg.2022.774165

**Published:** 2022-05-19

**Authors:** Muhammad Fareed, Aqeel Ahmad, Sri Sarah Maznah Mohd Salleh, Wan Shakizah Wan Mohd Noor, Mohd Faizal Mohd Isa

**Affiliations:** ^1^School of Business Management, Universiti Utara Malaysia, Sintok, Malaysia; ^2^Faculty of Management Studies, University of Central Punjab, Lahore, Pakistan; ^3^Centre of Excellence for Social Innovation and Sustainability, Universiti Malaysia Perlis, Sintok, Malaysia; ^4^Faculty of Applied and Human Sciences, Universiti Malaysia Perlis, Sintok, Malaysia

**Keywords:** human resource professionals’ effectiveness model, resource based-view theory, exploratory research approach, thematic analysis, telcom firms

## Abstract

Extant research primarily focuses on the driving factors of human resource (HR) professionals’ effectiveness in Telco firms of Pakistan. That is due to the need for HR professionals to be effective has risen in the 21st century for gaining sustainable competitive advantage. This research paper aims to examine the level of HR professionals’ effectiveness in Telco firms of Pakistan and explores the strategic functions and their elements, owing to which HR professionals can be effective in fulfilling their tasks and duties. Ten HR executives from five Telco firms were interviewed. The study finds nine key strategic functions along with their forty elements, which indicate the effectiveness of HR professionals. Successful implementation of these strategic functions and their elements offers Telco firms to sustain competitive advantage. Hence, it extends the resource-based view (RBV) theory by accumulating the additional determinants in the context of Pakistan. The research provides the insights for policy makers and practitioners, which explain the key strategic functions and substantial variables through which HR professionals can augment their effectiveness in sustaining competitive advantage.

## Introduction

At the moment, worldwide economies establish thoughtful challenges for human resources (HR) functions through forming a competitive environment in which firms must strive for sustainable competitive advantage. Likewise, in Pakistan, the developments in information and communication technology (ICT) have formed forceful competition due to customers and suppliers’ awareness (Imtiaz et al., 2015). In highly competitive telecom markets like Pakistan, suppliers are under persistent pressures to introduce fresh products and services, leaving them to be constantly creative and innovative to maintain their substance in the market (Hafeez et al., 2017). It demands a higher level of skillfull HR professionals in the organization to sustain long-term competitive advantage (Fareed et al., 2016c). To sustain competitive advantage and to perform critical roles more effectually, HR professionals must need to be effective. Ulrich et al. (2009) stated that, in the modern global economies, the business situations are turning more explosive, and competition is rising stronger each day. Thus, HR professionals are consistently expected to help organizations to compete in the marketplace, and, to do so, HR professionals are required to identify and acclimate to the current business fashions and challenges (Ulrich et al., 2008).

Ahmad et al. (2015) underlined that HR professionals are incapable to develop or execute good HR practices and strategies, which ultimately leads to employees’ dissatisfaction, and employees are unable to deliver what is expected from them. Likewise, the observation of weakness of the HR professionals’ attributes and competencies in Pakistan has also been observed and proposed as an impediment to organizational effectiveness (Sharif et al., 2011). As a result, the effectiveness of the HR professionals is under inspection in Pakistan (Sultan et al., 2012). Established on the prominence of the Telco industry, scholars underlined that the Telco industry has been the utmost hasty emergent industry in Pakistan (Fareed et al., 2020). It has experienced a lot of transformation in the last decade, comprising the evolution in technology, coverage of network, in addition to growing rivalry. These fluctuations eventually initiated the need of well-trained and effective HR professionals in the industry (Sultana et al., 2012). This is also due to Telco firms of Pakistan are not positioning HR at the center of their strategic missions and visions. In addition to that, they are not recognizing the significance of HR professionals as strategic partners of the organizations, while some of the prominent American firms have positioned issues related to HR at the center of their strategic mission and vision, and that is why they are able to sustain competitive advantage (Schuler, 1990; Ulrich and Lake, 1991). This indicates that the competencies and effectiveness of HR professionals need to be improved further, which will ultimately lead to the better organizational performance. Therefore, value-driven HR professionals in the 21st century should have the essential HR competencies to further enhance the level of their effectiveness (Ahmad et al., 2015), while previous researchers have studied the concept of HR professionals’ effectiveness (Wright et al., 2001a; Han et al., 2006; Ahmad et al., 2014, 2015) but through a quantitative research approach. The current study is first of its kind, which ratifies the factors and elements that contribute to the body of knowledge of HR professionals’ effectiveness using the subjective “qualitative” research approach.

In spite the fact that, Ahmad et al. (2014) also underlined that the research to determine the effectiveness of HR professionals being an area of academic research is quite at early stage in Pakistan. Thus, this study takes initiatives to examine the level of HR professionals’ effectiveness in the Telco firms of Pakistan by emphasizing the fact that the need for HR professionals to be effective has risen in the 21st century for gaining sustainable competitive advantage. Furthermore, scholars (Fareed et al., 2016a; Razzaq et al., 2017) proposed the need for upcoming researchers to examine HR practices using subjective viewpoints in the Telco firms of Pakistan. This is also supported by the Guest and Conway (2011), who signified the profound qualitative investigation for exploring HR effectiveness. Recent study by Kazmi et al. (2021) has explored the role of HR effectiveness as a mediator between transformational leadership and an organizational learning capability, suggesting researchers can also use qualitative and mixed approaches in the future. To address the aforementioned gaps in the literature, the current study pursues two main objectives. The first objective is to explore the level of HR professionals’ effectiveness in Telco firms of Pakistan. It further aims to find factors and elements that contribute to HR professionals’ effectiveness. Therefore, this study attempted to offer new avenues into the body of knowledge and practices of HR professionals’ effectiveness by measuring the level of effectiveness and, similarly, by exploring the factors and elements using the qualitative research approach, which impact this phenomenon in Telco firms of Pakistan.

The choice of the context of the study, the Telco industry of Pakistan, is significant since not much attention has been given on the issue of HR professionals’ effectiveness in this industry, considering the contribution of the industry in the country’s GDP (8.5%) (Ministry of Finance, Government of Pakistan, 2014–2013) and the significance of the industry as engine of the economy (PTA, 2013–2012). According to the Global Information Technology Report (2010-2011) given by World Economic Forum, Pakistan positions at number 1 on the internet and telephony competition as cited in Hafeez et al. (2017). The Telco industry is one of the promising industries of Pakistan as it has shown an unprecedented success in the past few years. The industry has similarly played an enormous role in economic development of the country in the last decade since massive investment has been approaching domestically and worldwide (Ministry of Finance, Government of Pakistan, 2014–2013). The industry has grown mainly due to trade and investment liberalization, favorable policies, and healthy competition. According to Business Monitor International (BMI), Pakistan has been ranked as the utmost favorable country for Telco development (Imtiaz et al., 2015). The Telco industry attracted more than USD 7.14 billion FDI in the last decade, whereas the Telco investments have been more than USD 15.14 billion, generating revenue of USD 29.62 billion along with the thousands of employment opportunities [PTA, 2013–2012, page 14, cited in Imtiaz et al. (2015)]. However, owing to the rapid advancement and intense competition in the industry, the need of competent and effective HR professionals is vital to the success of any organization in gaining and sustaining a competitive advantage (Kirwan and Birchall, 2006; Razzaq et al., 2017). Accepting the significance of this dynamic industry in Pakistan’s economy in addition to the question of the level of HR professionals’ effectiveness of Telco firms in Pakistan, this topic required to be empirically examined in the background of the proposition that HR effectiveness directly improves organizational performance (Schuler and Jackson, 2005; Ahmad et al., 2015). Hence, given the rapid growth of the Telco industry and the significance in the economic development of the country as well as due to the importance of Pakistan as an emerging market and the key geopolitical player in Central Asia (Hafeez et al., 2017), the current research opted to emphasize on this industry.

## Literature Review

Research on SHRM issues has grown rapidly over the last decade. The rising significance of personnel in the success of organization links with the growth of SHRM as a field of research worldwide (Boxall and Purcell, 2011). The ancient development of the HRM notion and practice can be seen through numerous standpoints. For instance, the development of HRM “as a specialized and scientific discipline, as an assistance to management, as a conflict management of political and economic values between employers and employees, and a rising movement of employees’ involvement, which is influenced by the industrial developments” (Schuler, 1990; Kabene et al., 2006). However, the function of HRM in the organization has changed consistently with the shifts in the economies, for instance, from agricultural to industrial to services and, currently, to information technology direction and attention (Ahmad et al., 2015). At the beginning, the HRM was considered a staff function and entailed the application and implementation of rules and policies only. With the development of economic orientation, scholars and practitioners inside and outside the organizations have come to realize that the employees are strategic resources in terms of the value that they offer to the organizations with the unique feature of being repositories of knowledge, skills, and capabilities. Nevertheless, if pertinent HR policies and processes are established and employed effectively, HR can make an extensive impact on organizational performance (Armstrong, 2011).

### Human Resources Professionals’ Effectiveness

Dessler (2010) stated that HR professionals typically perform their tasks and duties at the head office of an organization. However, while performing their tasks and duties, they can be either HR generalists or HR specialists. Both kinds of HR professionals need to perform an extensive amount of work experience in the discipline of HRM. As HR generalists, the HR professionals are expected to cope with all aspects and fundamentals of HR-related work, and they are obliged to have a wide range of competencies. Ulrich et al. (2008) proposed that an HR generalist is consigned to work with an organizational unit. The HR generalist is also responsible to engage and contribute to the strategic planning process and make sure those strategies would be implemented after using the HR professionals’ competencies, whereas Dessler (2010) addressed that HR specialists in bigger organizations usually oversee many different departments. Each department may be supervised by a manager who must be the HR specialist in the HRM function such as staffing, training and development, compensation, and performance appraisal. The organizations expect HR professionals would execute these functions efficiently and effectively.

Numerous researchers stated that HR professionals achieve their tasks in the organization and accomplish their responsibilities, which are associated with the organizational performance (Huselid, 1995; Wright et al., 2001b). Organizations, which are willing to enhance their HR professionals’ effectiveness, must emphasize on developing HR professionals’ competencies. Studies on HRM, in general, somehow ignored the impact of HR professionals’ competencies with the association between HR professionals’ effectiveness and performance (Ahmad et al., 2012). On the other hand, research has correspondingly absorbed several issues such as determining the managerial effectiveness, scrutinizing the progress of ensuring managerial effectiveness, and constructing models of managerial effectiveness (Sharif et al., 2011). Han et al. (2006) contended that HRM, by way of a management discipline, is in rather early stages in Asia paralleled to the western countries, and, as such, the HR professionals’ effectiveness has not been widely studied in Asia, including South Asia, in spite of the fact that ultimately developing nations are least focused on SHRM practices as compared to developed countries of the world (Razzaq et al., 2017). Subsequently, the current study put emphasis on the need to study HR practices in general and HR professionals’ effectiveness in focus on the Telco industry of Pakistan. It is suggested that if HR functions are successfully and effectively succeeded, it will reflect on the effectiveness of HR professionals, along with the organizations, and, subsequently, it can also be a source of competitive advantage since researchers stressed that HR effectiveness is strongly viewed as HRM contribution to an organizational performance (Ruel et al., 2007).

### Background of Human Resources Management in the Telco Industry of Pakistan

During the last decade, the global Telco industry has been persistent to grow, largely as a result of technological advancements and increasing trends of customization of smartphone acceptance. Similarly, the Telco industry of Pakistan has been developed primarily because of trade and investment liberalization, favorable policies, and robust competition (Fareed et al., 2016b). Fast expansion and aggressive rivalry in the industry are having a reflective consequence on how organizations cope with their strategies to attract and, most importantly, satisfy and retain their valuable employees. In the epoch of fierce competition, organizations in all industries of the Pakistan, especially in the telecom industry, are striving hard to satisfy employees and customers by supplying value-added services to stay in the competition (Razzaq et al., 2017). Therefore, the Pakistan Telco industry demands for having right people at the right job to keep track on employees’ effectiveness through innovative and modern HR practices to add in the value of shareholders. Yet, previous scholars have highlighted various HR issues in the Telco industry of Pakistan, which are due to HR professionals are weak in performing their roles and responsibilities effectively and efficiently, for instance, organizational commitment (Razzaq et al., 2017), lack of skillfull HR, retention of employees, compensation, and dearth in strategic roles of HR (Balagam and Fariduddin, 2008). Malik et al. (2012) have studied issues related to recruitment in the Telco industry of Pakistan. They have identified that environment, reference, selection standard, discrimination, and culture are among those issues and problems that have affected the recruitment process in the industry.

Furthermore, researchers advocated that, due to the intense competition in the Telco industry, organizations are setting extra burden on employees in sequence to strive for competitiveness, challenging demands, thrilling workloads, and physical working environments that cause job stress and which lead to reduced job satisfaction and, ultimately, turnover (Mansoor et al., 2011). An important issue of turnover in the Telco industry had also been observed by previous academicians (Rana et al., 2009; Shoaib et al., 2009; Hussain and Asif, 2012). They proposed that organizations are required to emphasize on how to develop a better salary plan and fringe benefits in addition to minimizing anxiety in the workplace for the purpose of reducing the issue of turnover. Rana et al. (2009) also argued that if employees willingly leave the organization, it is an immense failure for the Telco industry as it is costly to hire, train, and bring new replacements. Organizations would be able to enjoy and sustain the success until they deal with the problem of turnover efficiently and successfully. Therefore, it is important to recognize the background of turnover intent of employees prior to their decision to quit. In a such setting, HR professionals can play an enormously important strategic role by dealing with HR issues in their firms to enhance organizational productivity. Balagam and Fariduddin (2008) reinforced the argument that the Telco industry of Pakistan has not yet positioned its focus on HR professionals, and it is not recognizing the significance of HR professionals as strategic partners of the organizations, while some of the leading American firms have positioned issues related to HR at the center of their strategic missions and visions (Schuler, 1990; Ulrich and Lake, 1991). However, it is important to deal with HR issues for any organization in order to accomplish its strategic objectives and achieve competitive advantage.

### Theoretical Perspective

This study used resource-based view (RBV) of the firm as an underpinning theory by way of HR professionals as a source of sustainable competitive advantage for the firm. Conferring to RBV, organizations, which possess superior resources, might be able to perceive and execute distinctive strategies that competitors could not imitate easily (Barney, 1991). As rivals permit to replicate the superiority of physical and financial resources, experts and scholars have focused on the distinctiveness of HR as an aspect that can bring sustainable competitive advantage for a firm (Barney, 1991; Barney et al., 2001). In conformity with the RBV of the firm, it is required for organizations to classify, evaluate, and improve key HR professionals’ competencies permitted to achieve an adequate level of competitive advantage. According to RBV, it is HR professionals’ main responsibility to enable the organization’s corporate objectives through people since they are the ones who contribute to the organizational goal (Priem and Butler, 2001). It is essential for any resource to add value to the organization in accordance with RBV of the firm. However, that value must be rare, unique, and there should not be an adequate replacement for the resource (Barney, 1991). Firms invest in HR through training and development for the purpose of improving their potential to accomplish organizational tasks more effectively.

Inyang (2010) claimed that the essential notion of RBV of the firm is resource heterogeneity. This identifies that the resources that organizations have are unlikely to be identical, and they must encounter four requirements: They must be valued, exceptional, unique, and non-compatible. Moreover Snell et al. (1996) believed that HR comes across these four requirements, which, eventually, can create the robust source of competitive advantage for the firm. Ever since Barney (1991) drew the elementary theoretical model and measurement for the sources of sustainable competitive advantage, the RBV theory has become the most frequent philosophy in the discipline of SHRM, both in the expansion of the theory and the foundation for empirical research (McMahan et al., 1999). Consequently, this study intended to use RBV as a foundation to explore the level of HR professionals’ effectiveness in addition to pursue those factors and elements, which might lead to a sustainable competitive advantage of a firm in the shape of HR professionals’ effectiveness in the Telco firms of Pakistan.

## Research Methodology

The current study endeavors to explore the level of HR professionals’ effectiveness in Telco firms of Pakistan. It further aims to find factors and elements that contribute to HR professionals’ effectiveness in the context. To achieve this objective, a qualitative field study by means of semi-structured interviews has been deployed in this research. This included in-depth, open-ended questions, which followed the guidelines of Jacob and Furgerson (2012). The interview protocol has been developed by the researchers through extensive literature review, for instance (Huselid et al., 1997; Wright et al., 1998, 2001b; Mitsuhashi et al., 2000; Richard and Johnson, 2001; Han et al., 2006; Choi et al., 2010; Guest and Conway, 2011; Kim and Ryu, 2011; Ahmad et al., 2013; Ryu and Kim, 2013; Ahmad et al., 2015). HR professionals have been asked to rate themselves as per their perceptions of the level of effectiveness in core strategic functions, which were identified by the previous literature. Interview protocol is not only a set of questions but also a procedural guide for directing a qualitative researcher through the interview process (Jacob and Furgerson, 2012). To develop an interview protocol, the current research followed the interview protocol refinement (IPR) framework provided by Castillo-Montoya (2016). This framework basically comprises of a four-phase process, which includes (1) confirming interview questions align with research objectives, (2) creating an inquiry-based conversation, (3) getting feedback on interview protocols, and (4) piloting the interview protocol. However, following this framework has strengthened the reliability and validity of the interview protocol (see Appendix A) for the present study.

The face validity of the interview protocol is verified by the selected group of practitioners and academicians in Malaysia and Pakistan for the reflection of expert interpretations. These experts were asked to give their opinions if the interview protocol was appropriate to achieve the research objectives of the study. The experts endorsed that the interview protocol predominantly is relevant and appropriate to achieve the focal research objective of the study. Nevertheless, it is used as a guideline rather than a rigid structure. Using earlier research to guide interview questions means researchers have done a thorough review of the literature, which explains what previous scholars say about the phenomenon of interest. Knowing the past research leads to developing interview questions that are grounded in the literature yet differ from what previously studied. It also helps to narrow our focus and questions in ways that will create meaningful data. HR professionals were encouraged to explain their beliefs about the levels of their effectiveness and about the key drivers, which direct their effectiveness.

In the initial stage of data collection, emails asking for permission to conduct an interview were sent to each human resources department of the Telco firms in Pakistan. In the email, the research objective and methodology of the current study were briefly described. Once, the human resources department of each Telco firm responded, and HR professionals indicated their willingness to participate in the study; they were further contacted to fix the time and place. Primarily, the researcher has established contacts with the initial tier of HR professionals from Telco firms. Based on the responding behavior and the pattern of the participants, the study has used a convenient sampling technique. This is due to HR professionals were too resilient and busy in performing day-to-day responsibilities, although the HR professionals who had shown willingness to take part and who were convenient to reach were approached to be a part of the study. It should be noted that all the HR professionals involved in the field study were HR executives or managers of Telco firms who have enormous exposure and experience in HR within and outside the country. These HR executives are the key HR professionals who are responsible for all kinds of HR activities, and, similarly, they are engaged in the process of strategic HR planning inside the organizations. Thus, they are presumed as appropriate HR professionals for the interviews to gain insight into their experiences and to recognize the progressions.

Kadir and Noor (2015) stated that qualitative field studies could help society in understanding the complexities that business organizations face today. Furthermore, semi-structured interviews are usually constructed on a flexible procedure that offers a loose arrangement of open-ended questions to examine experiences and viewpoints of the participants (Pope et al., 2002). Morse (2005) also distinguished that, in the qualitative field study, all interviews must be considered equivalent, and the information gathered should be compared and analyzed item by item. Accordingly, Boyce (2006) specified that interviews permit a more comfortable atmosphere than a questionnaire to collect data as individuals feel more relaxed while having tête-à-tête with the researcher of their experiences in the workplace. It is believed that the most relevant characteristic of an interview is that individuals answer the questions honestly and confidentially.

As mentioned earlier, the study had applied a convenient sampling technique to ensure the robust and prompt information from the participants. This technique was the best available sampling technique, considering the eventful schedule of the participants and also owing to the lack of formal information of HR executives/managers in the websites of Telco firms or Pakistan Telecommunication Authority (PTA). Population of the study was not vibrant, and it was extremely difficult to find the proper/exact number of HR professionals working in the Telco industry of Pakistan. Hence, the study has used convenient sampling due to the subjective nature of the research, lack of a scientific figure of population, and because this technique is extremely useful when the researcher has limited resources, time, and workforce (Etikan et al., 2016). The general aim of sampling in qualitative research is to acquire information that is useful for understanding the complexity, depth, variation, and/or context surrounding a phenomenon rather than to represent populations as in quantitative research (Gentles et al., 2015). Zikmund (2003), similarly suggested that a convenient sampling technique can obtain extensive information quickly and effectively. Additionally, scholars (Cohen et al., 2000, p. 56; Gentles et al., 2015) recommended that, in “phenomenology qualitative research” less than ten participants would be enough if researchers intend to follow informants intensely. Phenomenology has been defined as a qualitative approach in which researchers aim to develop new understandings of existed human experiences, relying on first-person interpretations generally attained *via* interviews (Creswell and Poth, 2017). Cohen et al. (2000) also argued researchers cannot suggest the necessity of sampling decisions before the actual field study within hermeneutic phenomenology, as they stated: “*Exploring who shares a way of talking about a particular experience cannot be determined before the researcher enters the field”* (p. 54). Hence, the researchers intended to interview ten informants since intention was to follow each informant intensely rather than spread our attention over a large group of the participants.

The frequently suggested criterion for defining sufficient sample size in qualitative research is the point of saturation (Lincoln and Guba, 1985; Glaser, 1992; Morse, 1995; Charmaz, 2003; Merriam, 2009) as cited in Gentles et al. (2015). Guest et al. (2006) likewise suggested that, in qualitative research, mostly, sample size relies on the idea of “saturation” or the point at which no new information or themes are observed in the data. Moreover, they endorsed that, for most research initiatives in which the purpose is to comprehend shared perceptions and experiences among a homogeneous group, six to twelve interviews should be sufficient. Hence, interviews were conducted with ten HR managers/executives of the five Telco firms at their respective offices. Each interview took about 40 to 60 min, and it was recorded by a Micro-audio recorder (MP3) with the consent of the participants. HR professionals were selected on the substance of their willingness to provide the information by virtue of knowledge and experience. These HR professionals were proficient and well-informed about phenomenon of interest.

The most challenging thing in qualitative research is the method of data analysis and its formulation (Folkestad, 2008), likewise in the current research. Numerous tools and techniques were available; nonetheless, selection of tool(s) or technique(s) must be based on the objectives of the study. Since each interview was recorded through a (MP3) recorder, the data were apprehended by interpreting the interview tapes (MP3) into the interview transcripts. As stated earlier, each interview acquired about forty to sixty, consequently, interpreting the interview tapes (MP3) into the interview transcriptions took more than 2 weeks and more than 50 pages. After interviews were transcribed, content analysis has been recognized as an appropriate technique for analyzing the interview transcripts. And identification of the key themes and dimensions has been assembled by the way of detaining and getting the relevant elements and concepts of each variable from each participant. Content analysis was carried out on two stages “first-order and second-order” (Gehman et al., 2017). The first stage acts upon single-interview transcripts for identification of the concepts and key terms (first-order codes), whereas the second (2nd) stage acts upon cross interview transcripts for the validation of the concepts (second-order themes), which, in-depth, analyzes the similarities and differences of the factors and elements. Once all the first-order codes and second-order themes and dimensions have been accumulated, then we have the foundation of building a data structure. This, perhaps, is the most essential step in the entire research approach because it shows the progression from raw data to first-order codes to second-order theoretical themes and dimensions, which is an important part of demonstrating rigor in qualitative research. First-order and second-order terminologies seem to become progressively predominant in recent years in the qualitative field research (Gehman et al., 2017). Content analysis was done manually, and inductive as well as deductive reasoning was applied to classify the factors and elements. Therefore, the mapping of findings was done through a combination of inductive and deductive approaches of content analysis.

## Findings and Discussion

Data were collected from HR executives of Telco firms (Mobilink, Telenor, Zong, Ufone and Warid) through semi-structured interviews. These Telco firms are the top firms in Pakistan as per their market shares and subscribers. They hold 99% of the market share in the Telco industry as per the PTA (2013–2012). The researchers interviewed two HR managers/executives, each from Telenor, Mobilink, and Zong, while three from Ufone and one from Warid. This is due to, during the field study, Mobilink was in the process of consolidating Warid, which was officially publicized far along in PTA Annual Report (2016). Nonetheless, it was challenging to approach another HR executive from Warid; for that reason, the HR executive from Ufone was approached and providentially obtainable for the field interview. The aim behind was to exactly point out the experiences related to the performance of HR managers itself. Supporting the argument, Ahmad et al. (2014) encouraged forthcoming scholars to study HR managers in measuring HR professionals’ effectiveness. Table 1 specifies that the total number of HR professionals who were the part of qualitative field research is ten participants. Among which seven participants are HR executives, and one each participant is an HR manager, a manager of organization development (OD) and an HR director. All HR professionals have sufficient experience in the field of HR, which facilitated researchers to get thoughtful information and to gain insight into their experiences in the workplace. The HR professionals also had earlier worked in the HR domain prior to they had been employed by the current organization. The HR professionals have been recognized by their organizations to key HR positions after certain years of working experience and training. All of the ten HR professionals have completed their Master of Business Administration specialized in the human resources area. The HR professionals’ experiences and their qualifications symbolize that they are appropriate for the field interviews; therefore, they were reliable for the qualitative field research.

**TABLE 1:** Demographic information of the participants.

Participants	Company	Current Position	Working Experience	Highest Qualification
1	Telenor	HR Executive	11 years	MBA (HR)
2	Ufone	HR Executive	5 years	MBA (HR)
3	Zong	HR Executive	8 years	MBA (HR)
4	Mobilink	HR Executive	6 years	MBA (HR)
5	Warid	HR Operations Executive	6 years	MBA (HR)
6	Zong	HR Executive	6 years	MBA (HR)
7	Mobilink	HR Executive	7 years	MBA (HR)
8	Ufone	HR Manager	5 years	MBA (HR)
9	Telenor	HR Director	6 and half years	MBA (HR)
10	Ufone	Manager (OD)	5 years	MSC (HR)

Table 2 presents the insights of the participants into their levels of effectiveness in nine key strategic functions, which they perceive are essentials to execute if they want to be effective. Nevertheless, it has been affirmed that the level of HR professionals’ effectiveness is above the standards as their responses reveal higher proportions in all nine key strategic functions. These key strategic functions are expressed in below Table 2, accompanied by the level of effectiveness.

**TABLE 2:** The level of human resources professionals’ effectiveness.

	Participants									
Level of Effectiveness	P1	P2	P3	P4	P5	P6	P7	P8	P9	P10
	
Key Strategic Functions	Percentage (%)									
● Recruitment	90	75	85	90	85	90	80	90	50	70
● Responsiveness	80	60	95	80	90	90	90	80	80	65
● Communication	100	75	90	80	90	100	80	90	75	85
● HR Policies and Procedures	85	80	85	75	75	90	85	85	70	85
● Optimum HR Practices	90	50	90	90	80	90	80	75	80	85
● HR Responsibilities	85	50	90	85	75	100	60	80	50	60
● HR Roles	90	60	75	80	70	95	70	90	80	60
● Providing Career Plans	90	70	85	90	65	80	80	80	60	65
● Providing Support and Services	95	90	90	90	90	95	90	90	80	80
**Total Score**	**805**	**610**	**785**	**760**	**720**	**830**	**715**	**760**	**625**	**655**
**Average Score (Level of Effectiveness)**	**89.44**	**67.78**	**87.22**	**84.44**	**80.00**	**92.22**	**79.44**	**84.44**	**69.44**	**72.78**

*Average score = total score/nine strategic function; P1 - P10 (Participant 1 - Participant 10); P = Participant.*

The fable displays the level of HR professionals’ effectiveness and highlights the core strategic functions, which drive the effectiveness of HR professionals. Columns of the table illustrate the level of effectiveness in percentages, while rows represent the nine strategic functions, which HR professionals are obligatory to perform for the purpose of expanding their effectiveness. These strategic functions are recruitment of right candidates at the right time, responding to employees’ needs and demands, communicating timely information, developing HR policies and procedures, performing optimum HR practices, HR professionals’ roles, HR professionals’ responsibilities, providing career plans and providing support and services for the employees. Besides this, the level of effectiveness was established by the measurement of the average score (in percentage) given by each participant in every strategic function, whereas the average score was calculated as the total score divided by nine strategic functions (see Table 2). It is believed that there is no pragmatic approach or criterion available in previous research, which can lead us to understand the standards on the level of HR professionals’ effectiveness by following what we can postulate, that the level of effectiveness is higher or not. This is since previous researchers (Wright et al., 2001b; Han et al., 2006; Choi et al., 2010; Ahmad et al., 2014, 2015; Fareed et al., 2016b) who have studied the concept of HR professionals’ effectiveness in various contexts have not established any yardstick through which we can understand the level of effectiveness related to HR professionals. Consequently, this is originated by the current study from the responses of the participants itself, who verified the standards of being effective and not effective. Explicitly, their effectiveness levels would be higher if they are 80 per cent and above, whereas anything in between 60 per cent and 80 per cent would be an acceptable level, and less than 60 per cent reveals that HR professionals are ineffective in performing their strategic functions. This is supported from the responses of HR professionals:

“… *I can say*, *due to those systems and portals*, *we turned out to be highly effective in recruiting potential candidates*, *and I would give it 80%.” (HR Executive A, Warid)*

“…*all the HR procedures are approved from the top management*, *and*, *sometimes*, *management is not even involved in it*… *thus, I think we still need to be effective in developing HR procedures*; *therefore, I would rate it 50%.”(HR Executive A, Warid)*

“…*being an HR professional, employees look forward to support from us*, *and we have been doing that quite effectively that is why I would say 85%.” (HR Executive B, Mobilink)*

“…*we have to perform our roles effectively*, *which are stated in the book. And as I mentioned earlier*, *performance management system (PMS) facilitates us to fulfill the expectation of our employees*, *so I will rate it 80%.”(HR Executive A, Mobilink)*

Nonetheless, this study has established the yardsticks for the upcoming research for the level of effectiveness of HR professionals by the responses from HR executives of Pakistan’s Telco firms. Consequently, Table 2 identified that most of HR professionals are effective in performing every key strategic function, which consequently establishes their levels of effectiveness are higher. In addition to that, we can interpret from the Table, among all the participants; Participants 1, 3, 4, 5, 6, 7, and 8 are exceptionally effective by means of their average scores are between the range of 79 and 92. These HR professionals are effectively performing each key strategic function comprehensively. Conversely, Participants 2, 9, and 10 are in the acceptable level of effectiveness as their average scores are 67.78, 69.44, and 72.78, respectively. These HR professionals are partially effective in performing key strategic functions. On the contrary, none of them have been found less effective as per the standards set by this research from the responses of HR executives.

In addition, below Table 3 presents the essential components of key strategic functions, which further explain that the HR professionals are effective because they are effectually performing these components to accomplish key strategic functions. Hence, these components form the list of tasks, duties, and responsibilities of each HR professional, which he or she has to accomplish to produce desired results. Each row of the Table presents the components of key strategic functions, which drive HR professionals’ effectiveness, while columns of the Table identify the responses from each participant. The √ sign represents the implementation of each component.

**TABLE 3:** Elements/dimensions and strategic functions for human resources professionals’ effectiveness.

	Participants									
Elements/Dimensions of Strategic Functions	P1	P2	P3	P4	P5	P6	P7	P8	P9	P10
**Recruitment**										
● Strategic HR Planning	**√**	**√**	**√**	**√**	**√**	**√**	**√**	**√**	**√**	
● Well Established Staffing Process	**√**	**√**	**√**	**√**	**√**	**√**	**√**	**√**		**√**
● Staffing Performance Targets	**√**		**√**	**√**	**√**	**√**	**√**	**√**		
● Strategic External Recruitment	**√**	**√**	**√**	**√**	**√**	**√**	**√**	**√**	**√**	**√**
● Strategic Internal Recruitment	**√**		**√**	**√**	**√**	**√**	**√**	**√**		**√**
**Responsiveness**										
● Employees’ Well Being	**√**	**√**	**√**	**√**	**√**	**√**	**√**	**√**	**√**	**√**
● Quick Response	**√**		**√**	**√**	**√**	**√**	**√**	**√**	**√**	
● Effective Employee Relations Channels	**√**	**√**	**√**	**√**	**√**	**√**	**√**	**√**	**√**	**√**
**Open Communication**										
● Sustainable Competitive Advantage	**√**		**√**	**√**	**√**	**√**	**√**	**√**	**√**	
● Selective but Transparent, Honest and Consistent Information	**√**	**√**	**√**	**√**	**√**	**√**	**√**	**√**	**√**	**√**
● Communication Tools	**√**	**√**	**√**	**√**	**√**	**√**	**√**	**√**	**√**	**√**
● 360° and Continuous Feedback	**√**		**√**	**√**	**√**		**√**	**√**	**√**	**√**
**HR Policies and Procedures**										
● Appropriate Feedback Channels	**√**	**√**	**√**	**√**		**√**	**√**	**√**	**√**	
● Annual Revision of Policies and Procedure	**√**	**√**	**√**	**√**		**√**	**√**		**√**	**√**
● Employee Relations and Benefits	**√**	**√**	**√**	**√**	**√**	**√**	**√**	**√**	**√**	**√**
● Employees’ Engagement	**√**	**√**	**√**	**√**	**√**		**√**	**√**		**√**
● Employees’ Empowerment and Flexibilities	**√**		**√**	**√**	**√**	**√**	**√**	**√**	**√**	**√**
**Optimum HR Practices**										
● Annual Strategic Planning Alignment via Practices and Procedures	**√**		**√**	**√**		**√**	**√**	**√**	**√**	**√**
● Proactive HR Practices	**√**		**√**	**√**		**√**	**√**	**√**	**√**	**√**
● Competent Team Formation			**√**	**√**	**√**	**√**	**√**	**√**	**√**	**√**
● High Employee’s Commitment and Devotion	**√**		**√**	**√**	**√**	**√**	**√**	**√**	**√**	**√**
**HR Responsibilities**										
● Proactive HR Approach	**√**	**√**	**√**	**√**		**√**	**√**	**√**	**√**	**√**
● Empowerment of Team Leader/Manager	**√**		**√**			**√**	**√**	**√**	**√**	**√**
● Employees’ Needs Fulfillment	**√**	**√**	**√**	**√**	**√**	**√**	**√**	**√**	**√**	**√**
● Performance Management System	**√**	**√**	**√**	**√**	**√**	**√**		**√**	**√**	**√**
**HR Roles**										
● Strategic Business Partnership	**√**		**√**	**√**	**√**	**√**	**√**			**√**
● Formal and Informal Communication Sessions	**√**		**√**	**√**	**√**	**√**	**√**	**√**	**√**	
● Key Performance Indicators	**√**	**√**	**√**	**√**	**√**	**√**	**√**	**√**	**√**	**√**
● Effective Management of Employer – Employee Relationships	**√**		**√**	**√**	**√**	**√**	**√**	**√**	**√**	
**Providing Career Plans**										
● Organizational Support	**√**	**√**	**√**	**√**	**√**	**√**	**√**	**√**	**√**	**√**
● Personal/Individual Development Plan	**√**		**√**	**√**		**√**	**√**	**√**	**√**	
● Effective Performance Management System	**√**	**√**	**√**	**√**	**√**	**√**		**√**	**√**	**√**
● Talent Management Program	**√**		**√**	**√**	**√**	**√**	**√**	**√**	**√**	**√**
● Annual Training Programs	**√**	**√**	**√**	**√**	**√**	**√**	**√**	**√**	**√**	**√**
● Career Development Opportunities	**√**	**√**	**√**	**√**		**√**	**√**	**√**	**√**	**√**
**Providing Support and Services**										
● Cultural Values and Beliefs	**√**	**√**	**√**	**√**	**√**	**√**	**√**	**√**	**√**	**√**
● Strong Management Support	**√**	**√**	**√**	**√**	**√**	**√**	**√**	**√**	**√**	**√**
● Effective Communication	**√**		**√**	**√**	**√**	**√**	**√**	**√**	**√**	**√**
● Reliance on Team Members Support	**√**	**√**	**√**	**√**	**√**	**√**	**√**	**√**	**√**	**√**
● Effective Feedback Channels	**√**	**√**	**√**	**√**	**√**			**√**	**√**	**√**

Driving from Table 3, we can postulate that every HR professional in the Telco firm executes these signified elements of core strategic functions to achieve effectiveness. These vital elements have been affirmed as of the field interviews from the HR professionals of Pakistan’s Telco firms. It has been specified that these elements are purely the subjective insights of HR professionals. To improve HR effectiveness, HR professionals are required to execute core strategic functions and their elements. Besides, Table 3 validates all HR professionals recognize that each element is considerably important for HR professionals to be effective. However, these elements are the absolute forms that drive HR professionals’ effectiveness.

First, the core strategic function is recruitment, in which HR professionals perform strategic HR planning for the assessment of staffing needs. Similarly, they have very well-structured/established staffing processes in which they attempt to realize staffing performance targets. Once HR professionals identify the staffing needs, they can either look for a talent pool within their organizations (internal recruitment) or they can go for external recruitment. HR professionals endorse these elements as the vital elements to be effective in recruitment. For instance, HR professionals indicate that:

“…*when we make the annual strategy or plan for any department*, *there is another exercise which is strategic workforce planning for the year in which we define the staffing needs or staffing targets. Besides this*, *we have a very structured talent interview session for all employees in which we discuss with each individual along with his/her strengths and weaknesses*, *and then we define the development plans for that person*…*” (HR Executive A, Telenor)*

“…*in recruitment we*, *have certain KPIs*, *which we must follow, and we have dashboards going on every month*, *and those dashboards are being seen at the management level*, *that how we are performing. In terms of talent acquisition, we have KPIs of filling each position within a certain period, so we have to be effective in recruiting candidates at the right time*…*” (HR Executive A, Mobilink)*

“…*now*, *technology is moving so fast, and we are moving from 3G to 4G in Pakistan*, *and the telecom industry is so vibrant in the country. So, it is easy for us to find technical people in the telecom field. Because we can hire good human resources from our competitors by offering them higher packages*…*” (HR Executive A, Zong)*

“…*Well, while recruiting any applicant*, *we use behavioral skills*, *and we do have panel interviews during the recruitment process. Furthermore, any applicant is being assessed by the panel on different descriptive behaviors, and he or she knows what key aspects he or she needs to investigate. Therefore, we are very particular about it*…*” (HR Executive B, Ufone)*

“…*there is a complete succession planning process as well. Start from the workforce planning, moving toward, we do have very strong employee value proposition (EVP). And our value proposition defined on the parameters of which Telenor would like to attract external or retain the internal talent. We also do have recruitment HR information system (HRIS). Overall, the recruit system we start from the need analysis*…*” (HR Executive A, Telenor)*

The second strategic function is responsiveness. HR professionals articulate that responsiveness is the fulfillment of needs of the employees, i.e., whether HR professionals respond adequately to provide services that meet internal customer needs. They have approved three elements of responsiveness, which are employees’ well-being, quick response, and effective employee relations channels. It anticipates that HR professionals lean toward effectively responding to the needs of their employees. Perhaps, effective responsiveness embraces within the key performance indicators (KPIs) of the HR department, which HR professionals believe is an integral part of their culture. In fact, it is an open-door policy, where each employee can walk in and share his or her thoughts and ideas with the top management. In view of that, most of Telco firms are implementing the business partnership model; the aim of which was to bridge the gap between employees and top management/employer and work very closely with the business. Consequently, HR professionals in Telco firms of Pakistan are highly responsive to the needs of their employees. It is correspondingly maintained from the responses of HR professionals:

“…*I think*, *because with the company that is built around the people. There are a lot of activities on people development*, *and a lot of benefits have been provided for the employees. There are companies that are result oriented, but we are people oriented*, *and we take care of our employees and their families, and it has become a part of our culture*…*” (HR Executive B, Telenor)*

“…*Ufone stands for its human assets, so we do not compromise in responsiveness of fulfilling an employee’s needs*, *and that is something that includes in our KPI*…*” (HR Executive B, Ufone)*

“…*I believe we have effective employee relations’ channels*, *which are highly responsive to fulfill the needs of employees*…*” (HR Executive C, Ufone)*

“…*Starting from the position advertisement till the on boarding of the candidate, we know what the responsibility of the line manager is and what is the responsibility of an HR professional. So, this gives us a clarity in terms of line, in terms of HR that where we are standing and at the end of each month, these reports are being presented to management, higher management*, *and line managers. So, they get the visibility of how we are performing, where are the areas we need to improve in. So, this is how we are building the relationship with the employees*…*” (HR Executive B, Mobilink)*

Moving forward, third core strategic function is open communication. The Table certifies that there are four elements that make HR professionals become effective in communicating with their co-workers and employees, which are sustainable competitive advantage; selective, transparent, honest, and consistent information; communication tools; and 360 degrees and continuous feedback. According to the interviewed HR professionals, vulnerable communication and timely information are the most vital parts of any organization and/or even for HR professionals since the HR manager acts as a change agent. Vulnerable communication contains selective, transparent, honest, and consistent information sharing. Moreover, HR professionals’ performance evaluation (KPIs) is based on how effectively they communicate, which means right information with the right person at the right time. Accordingly, HR professionals engage in various communication tools for effective communication, such as groups, portals, helplines, verbally, and employees’ logbooks. Most importantly, HR professionals believe 360 degrees and continuous feedback through open communication can assist them in achieving individual and organizational goals effectively, and it can be so critical for an organization to advance its sustainable competitive advantage. This is sustained by the responses of HR professionals:

”…*Communicating timely information can be so critical for an organization for sustaining its competitive advantage. However, Ufone has a dedicated communication team*, *which makes sure that timely information is flowing throughout the organization*…*” (HR Executive C, Ufone)*

“…*We communicate with employees through different portals, tools, and groups. And I think it is very important to communicate right information at the right time with our employees, so we are so watchful in communicating with employees*…*” (HR Executive A, Warid)*

“…*Every policy we change or every change in the role will be announced in the company. And if the policy changes, then*, *we will have a policy session with our employees in which we share with them the information that is required. We share with them verbally, through portals*; *they can ask from our helpline and even they can see on the book*…*” (HR Executive A, Zong)*

“…*I have a very important responsibility of maintaining a good relationship with employees in term of on-going communication, where it is required, because*, *sometimes*, *if you communicate too much with your employees*, *then it creates chaos. So, it must be a calculated communication that what is required, where it is required*, *and why it is required. For example, in the transition phase*, *the most important thing was communication*, *that why this change is required. However, we have done it quite well*…*” (HR Executive A, Mobilink)*

“…*It is not about the communication*; *perhaps, it is more like using the right words at the right time with the right audience. So, it is the philosophy behind the change management and communication, and Telenor strongly believes that there is nothing to hide* - *whatever the thing is*, *we have to be honest with our employees*; *whatever the case is we*, *must be transparent and consistent with our employees. Our mission and targets are always to get in touch with our employees at the right time with right communication*…*” (HR Executive A, Telenor)*

The Table exhibits that the fourth strategic function is developing HR policies and procedures to support the company’s vision, mission, and values. HR professionals permit six elements of effective HR policies and procedures; the contemplation of which they should consider while developing HR policies and procedures. These elements are appropriate feedback channels, annual/semi-annual revision of policies and procedures, employee relations and benefits, employees’ engagement, and employees’ empowerment and flexibilities. It is important to note that HR professionals must use appropriate feedback channels to communicate HR policies with their line through which they can articulate the company’s vision, mission, and business plan. Moreover, HR policies and procedures need to revamp yearly or two times a year for the growth of employees’ relations and benefits. HR professionals should develop such HR policies and procedures, which focus on development and engagement of employees with the sense of physical, cognitive, and emotional commitment and involvement to contribute to organizational success. Finally, yet importantly, HR policies should reinforce employees’ empowerment and flexibilities with a view to expand innovation and productivity. As HR professionals state that:

“…*I think policies are so vital for the company through which we can build our relations with employees, and we can show our values and tell our employees how to behave within the company*…*” (HR Executive A, Warid)*

“…*Overall, in employees’ policies*, *there are a lot of flexibilities, benefits, and empowerment to the employees to perform their duties as per the policies. We have the structured manuals, and our processes are documented. Our company is the first in whole Asia*, *which ISO9001-2008 certified with respect to our procedures and policies. Our systems and procedures are ISO9001-2008 certified*…*” (HR Executive A, Telenor)*

“…*There is a lot of stiffness in HR policies at Ufone. A great deal has been given to employees’ benefits, empowerment, and engagement to get the greater satisfaction level of employees. And*, *furthermore*, *we provide the solutions of employees’ problems and issues so they can perform better*…*” (HR Executive C, Ufone)*

“…*We compare our policies with our competitors, and we feel that our staff gets maximum benefits as compared to our competitors. However, we revise our policy after every 6 months*…*” (HR Executive A, Zong)*

In performing optimum HR practices, there are four elements that have been recognized by the HR professionals. These elements are annual strategic planning alignment *via* practices and procedures for allocation of goals, proactive HR practices, competent team formation to monitor HR practices, in conjunction with high employee’s commitment and devotion. HR professionals consider that optimum HR practices speed up employees’ effectiveness and the firm’s productivity, and it also helps firms to succeed employees’ commitment and devotion. Nonetheless, those HR practices must be aligned with the company’s annual strategic planning and requirements. Notwithstanding this, HR professionals must form competent functional teams who should act proactively upon each HR practice. HR professionals ratify all the elements in performing optimum HR practices, which is the fifth strategic function. For example, HR professionals specify that:

“…*Each year*, *we start a New Year with looking at our progress from the last year, and we make our plans for the New Year*, *which is the called strategic planning part. We do the planning because we get the objectives from the top management*, *and we know what the challenges are for this year, what are the budgets, what are the men power requirements, demands and supplies in terms of staffing. So, we work proactively and work on these practices*…*” (HR Executive B, Mobilink)*

“…*In performing HR practices, we are quite flexible. We do strategic planning each year*, *and*, *accordingly*, *we develop our HR practices*, *and I believe we are quite effective in it as our employees are highly committed and devoted to the organization*…*” (HR Executive B, Ufone)*

“…*There is always room for improvement. Trends are evolving, and there are some practices related to talent management or employees’ commitment*, *which are flowing from the Telenor group*, *but there are some that flows from the HR department because local context needs to be very relevant, so I think we still have some room to cover*…*” (HR Executive B, Telenor)*

“…*We start our year with strategic planning*; *the objective of which is to investigate improvement areas and plans for New Year. We do the strategic planning part at the start of the year to achieve those objectives*, *which have been set by the top management. We work on those plans and improvement areas effectively*…*” (HR Executive A, Mobilink)*

“…*I am the only HR business partner in the central region. And the central region is so wide, and many employees come under this region, so I have to be proactive in developing HR practices*…*” (HR Executive B, Zong)*

“…*We are effective in HR practices because we have a highly competent team that looks after all the issues related to this matter*…*” (HR Executive C, Ufone)*

Performing HR responsibilities to meet the expectation of employees is the sixth strategic function that HR professionals should achieve to augment their effectiveness. HR professionals have to execute all HR activities in performing HR responsibilities; more specifically, HR professionals are responsible for one-window operation to maintain a good relationship with employees in addition to the business. They have documented four elements, which are important in fulfilling their responsibilities, which are a proactive HR approach to look after HR activities, empowerment of a relevant team leader or manager to fulfill employees’ expectations, contentment of employees’ needs, and performance management system. To facilitate employees’ expectation of pleasing their needs, HR professionals are responsible to adopt a proactive HR approach through performance evaluation system, which facilitates them to empower their employees. This was supported by the responses of HR professionals:

“…*There are two responsibilities of HR*: *first is where HR directly manages the employees’ expectation*, *and second is where HR empowers the relevant team leader/manager who manages the expectation of his or her employees. Certainly, there are few scenarios where HR directly intervenes and manages the expectations. There are most likely individual cases, but*, *most of the times, we enable or empower leaders with all right HR information so they can stand in front of their teams and manage their teams’ expectations as per the guidelines.”(HR Executive A, Telenor)*

“…*We do have some extra funds at the back end, and we tell our employees we are fulfilling their needs in different ways, but we cannot fulfill their expectations always the way they want*…*” (HR Executive A, Warid)*

“…*As I specified before*, *we are extremely responsive to fulfill the needs of employees*, *and that is something related to what employees are expecting from us in a way how we act on fulfilling their needs. However, I believe we must be very proactive in meeting their expectations as it comprises in our KPI*…*” (HR Executive B, Ufone)*

“…*For expectation*, *the part we use a system called performance management system (PMS) in which my performance is being evaluated and my feedback is not only coming from my line managers*; *it is like 360 feedback in which employees also give their inputs. So, I think there is no room for error*, *and if we are not doing our job, then we are bound to be held responsible*…*” (HR Executive A, Mobilink)*

HR professionals acknowledge four elements within HR roles, which are strategic business partnership, formal and informal communication sessions such as “casual tasks, email correspondence, informal chats,” meeting the key performance indicators (KPIs) and effective management of employer and employee relationships. HR professionals drive a higher level of effectiveness by executing above-stated elements. The HR role has evolved in the recent decade; it is no more kind of a transactional role; now, it is more a like strategic partner of the business. HR professionals must work very closely with their businesses to maintain a good relationship between management and employees. Basically, being strategic business partners, HR professionals help their leaders and businesses as well as their people from the strategic points of view so they can add value to the business. This partnership model assists HR professionals to work closely with their business and bridge the gap between management and employees. It is like transferring the emotions of employees to the higher management and then going back with solutions or taking back the leadership team with a solution to the lower employees. Basically, the purpose of introducing the HR business partnership model is that Telco firms want HR professionals to be more visible in terms of adding value to the business and in terms of their effectiveness since the HR business partnership model is grounded on the key performance indicators (KPIs). HR professionals acknowledge:

“…*I must translate the business sense to the employees and the voice of employees into the business and top management. So that is something that we tend to keep on doing.” (HR Executive A, Ufone)*

“…*Our roles are KPI based*, *so I have to be effective in fulfilling my roles*…*” (HR Executive A, Zong)*

“…*With the help of right information, processes, transparency, consistency, continuous engagement, and emotional touch with the employees, we do manage the expectation of employees*, *and that is our role*…*” (HR Executive A, Telenor)*

“…*Our roles are defined in the book through which we effectively manage the expectations of employees*…*” (HR Executive A, Warid)*

*“We have a highly responsive employee relation’s function to measure employees’ opinions, and that is why HR professionals are effective in their roles to meet the expectations of employees*…*”(HR Executive C, Ufone)*

The eighth strategic function is providing career plans for the employees. It has been underlined that there are six elements in providing career plans, which have been recognized by the HR professionals. Starting with organizational support, which is the most vital element in proving career plans or development opportunities, the subsequent element is personal development plans or individual development plans for top employees. Moving forward, Telco firms effectively accustom performance management system (PMS) to identify high performers (HiPos) to whom they provide career development opportunities. The fourth element is the talent management program by means of which organizations identify those employees who have potential to develop and even key positions which organizations would like to fill through succession planning. The fifth and sixth elements are annual training programs as per training calendar and career development opportunities for HiPos. It is in line with the responses of HR professionals of Telco firms of Pakistan, who indicated that:

“…*We provide career plans for those employees who are high performers (HiPos) as the telecom industry is the highly saturated industry*, *and we must support high performance in our organization. However, we identify HiPos in the telnet acquisition plan*, *and we see their potentials to grow*, *and*, *if they are ready to grow*, *then we do provide them career plans*…*” (HR Executive C, Ufone)*

“…*We have regular training programs and a training calendar that has been given to all the employees. In development*, *we have a 3E model*: *experience, education*, *and exposure. For education*, *we do have training programs*, *and*, *for experience and exposure*, *we change the roles of senior employees. So, there is whole development philosophy in place*…*” (HR Executive B, Telenor)*

“…*For career planning we have a talent management program in which we identify high performers (HiPos). Through this program*, *we identify those employees who have potential to grow and those positions which we must fill. Employees who are identified as HiPos* - *we have career plans for them, we have developmental plans for them, and we work on these plans throughout the year*…*” (HR Executive A, Mobilink)*

“…*Every year*, *we assess our employees through performance management system (PMS). We evaluate our employees based on KPIs. And employees set their KPIs/goals by themselves with the help of their line managers. And then*, *we decide to whom we should provide with the career plans and who should be promoted and who should be demoted*…*” (HR Executive A, Warid)*

And the last but not the least, the Table reveals the ninth strategic function is providing support and services in which HR professionals have attributed five elements. Cultural values and beliefs, strong management support, effective communication, reliance on team members’ support, and effective feedback channels are the five elements which make HR professionals effective in providing support and services for their employees. It is believed that, without the support of top leadership along with the HR professionals, employees cannot achieve their targets set by the organization. And employees always look forward to the necessary support from HR, although it embraces an additional key performance indicator (KPI) of HR professionals. Therefore, for being effective, HR professionals must provide support and services for their employees. As HR professionals certify that:

“…*It is about the culture*, *and Telenor believes our culture is our strength. We believe in empowerment; we believe in showing more trust to the employees. So, whatever the support is needed to the employees from the company’s leadership, we do provide them, whether this is with respect to work life balance or financial assistance or certain certification. Leadership is quite supportive at the Telenor group*…*” (HR Executive A, Telenor)*

“…*We have a strong and responsive employee relation’s function as I have stated before*; *this ER function is proactive in addressing employee concerns through a strong feedback channel*…*” (HR Executive C, Ufone)*

“…*Supporting our colleagues or our subordinates is very important at Mobilink. When we work in teams*, *so we are reliant on each other’ support*, *and*, *without it*, *I think we cannot complete our tasks effectively. Consequently, being HR professionals, employees look forward to support from us*, *and we have been doing that quite effectively*…*” (HR Executive A, Mobilink)*

“…*Whatever employees require my side, I give it to them. If I cannot really deliver it, I tell them that I cannot. I do not make them believe that I can and then not deliver on it. So, I provide support and services wherever I can and to whatever extent I can. Whichever is possible for me, I fight for them where I believe that I need to fight for them*…*” (HR Executive A, Ufone)*

“…*Without top management or HR support*, *employees cannot perform better. I believe support is always needed in any organization*; *it belongs to any industry. As the telecom industry is so a vibrant industry, however, this dynamic diligence increases the importance of providing support for the employees*…*” (HR Executive B, Ufone)*

**FIGURE 1:**
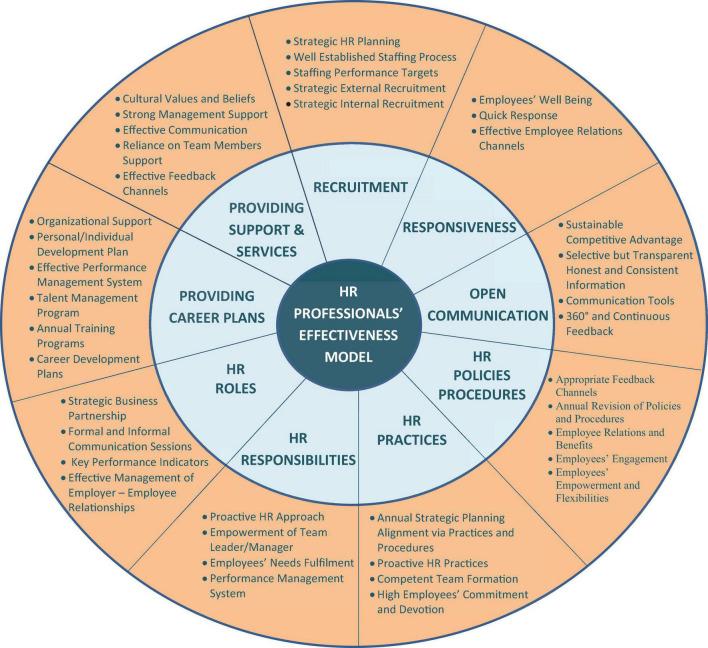
Summary of the findings. (HR professionals’ effectiveness model).

Eventually, in the view of Tables 1, 2, we can conclude that the level of HR professionals’ effectiveness is higher in the consequence of their being actively involved in all elements and dimensions of each strategic function (see Figure 1), which are essential for the comprehension of effectiveness. Figure 1 is the summary of the findings of the current study.

This research empirically validated the level of HR professionals’ effectiveness in the Telco industry of Pakistan. Findings reveal that effectiveness of all HR professionals partially or completely is on the higher level (above 80%) in each Telco firm (see Table 2). These findings are consequently owing to HR professionals are effectually carrying out key strategic functions, which assist them to augment effectiveness in performing their duties and responsibilities. Those nine strategic functions as we can see from the first-level HR effectiveness model in Figure 1 are: (1) recruitment of right candidates at the right time, (2) responding to employees’ needs and demands, (3) communicating timely information, (4) developing HR policies and procedures, (5) performing optimum HR practices, (6) HR professionals’ roles, (7) HR professionals’ responsibilities, (8) providing career plans for the employees, and (9) providing support and services. Additionally, qualitative findings also exposed forty vital components of the strategic functions in the second level of the HR effectiveness model in Figure 1. It is believed that HR professionals’ effectiveness is radical because of effective execution of the strategic functions and their components. These vital components are entirely the intuitions and perceptions of HR practitioners of Telco firms of Pakistan. Nevertheless, HR practitioners’ insights are enormously critical to a way that the vital components are extremely important for HR professionals’ effectiveness. The Telco industry is a very dynamic and the most rapidly growing industry in Pakistan (Ahmad and Ahmad, 2014; Khan et al., 2016). Due to the dynamics of this industry, it demands extremely well-trained and highly effective HR professionals (Hussain and Asif, 2012; Sultana et al., 2012) for attaining a sustainable competitive advantage (Barney, 1991; Wright et al., 2001a).

## Conclusion

As the Telco industry continues to grow at a hasty pace, the significance of HR professionals for being effective rises to sustain the competitive advantage. This study intended to explore the factors and elements that contribute to HR professionals’ effectiveness in the Telco firms of Pakistan. Additionally, it has also assessed the level of HR professionals’ effectiveness. Findings of the study demonstrate that HR professionals are effective because they actively engage in all the strategic functions, which are essential to enhance HR effectiveness. It is imperative to understand the significance of each element within the strategic functions of HR professionals’ effectiveness. This study concluded with vital exploration of nine strategic functions and their forty dimensions through semi-structured interviews from HR professionals of Telco firms of Pakistan.

## Theoretical and Practical Implications

This research paper contributes to the concept of HR professionals’ effectiveness by adopting a qualitative research approach through measuring the level of their effectiveness, which is believed to be an under-researched area in Pakistan since earlier studies such as Khan et al. (2011), Ahmad et al. (2014, 2015) inquired the level of core competencies and effectiveness of HR professionals in the banking sector of Pakistan, while strongly encouraging future researchers to extend their models to other industries or sectors of Pakistan such as Telco industry. Established on the prominence of the Telco industry, Sultana et al. (2012) emphasized that this industry is the swiftest and growing industry in Pakistan, which has experienced a lot of transformation in last decade. Furthermore, abovementioned scholars suggested that HR professionals’ effectiveness needs to be enhanced further, which will ultimately lead to the better organizational performance and effectiveness. Consequently, the preset study offers the level of HR professionals’ effectiveness as a point of reference for the forthcoming researchers. It further contributes to the theory and practice by exploring nine key strategic functions and their forty dimensions, which lead to the development of HR professionals’ effectiveness. The study adds to the resource-based view (RBV) theory by stressing that HR professionals can advance a sustainable competitive advantage for a firm by executing core strategic functions and their dimensions. However, these factors and variables, precisely, are the subjective standpoints of practitioners from Telco firms of Pakistan. Previous researchers have studied the concept of HR professionals’ effectiveness (Wright et al., 2001b; Han et al., 2006; Ahmad et al., 2014, 2015) through a quantitative research approach; however, this study is the first of its kind, which ratifies the factors and elements that contribute to the body of knowledge of HR professionals’ effectiveness using a subjective research approach. Additionally, none of the previous studies yet examined the level of HR professionals’ effectiveness. The study presents HR professionals’ effectiveness’ levels as a benchmark for future studies. Therefore, this study would be a significant initiative in the development of the levels of HR professionals’ effectiveness. The study is also important for HR practitioners and organizations as it provides suggestions and guidelines by the means of the HR effectiveness model to sustain the competitive advantage of a firm. The research paper offers implications for HR practitioners worldwide, in general, and of Pakistan, in specific, to assume a resilient and more active role in revealing issues embedded in HR professionals’ effectiveness, which are often underemphasized in the organization. Our study offers more general implications to the global HR practitioners due to its generic nature since the findings of the study come up with the HR effectiveness model, which previous studies are unable to identify. However, the research is just a beginning to establish a more comprehensive HR effectiveness model, which is a significant gap in SHRM and management literature. Findings similarly provide the level of an effectiveness scale as a guideline for HR practitioners to set the effectiveness targets for organizational HR. The findings of the study might encourage organizations to start positioning their HR at the center of the strategic mission and vision as HR is the most valuable asset for any organization.

## Future Directions

Future research should examine the long-established conceptual research model “shaped by the current study” through a quantitative research approach by testing a hypothesis. Forthcoming studies can test this model by applying a structure equation modeling (SEM) approach. Additionally, upcoming studies can possibly extend the qualitative sample size to make the findings more appropriate and comprehensive as the findings of this study cannot generalize to other contexts. Future researchers might also include HR practitioners from other sectors or industries as well as obtain data from different levels of employees to point out the experiences related to the effectiveness of different levels of employees.

## Data Availability Statement

The original contributions presented in the study are included in the article/supplementary material, further inquiries can be directed to the corresponding author/s.

## Ethics Statement

Ethical review and approval was not required for the study on human participants in accordance with the local legislation and institutional requirements. Written informed consent from the patients/participants or patients/participants legal guardian/next of kin was not required to participate in this study in accordance with the national legislation and the institutional requirements.

## Author Contributions

MF contributed to the article drafting and literature review. AA did the data collection and interviews. SS contributed to the discussion and conclusion. WN did the analysis and proofreading. MI performed the methods and analysis. All authors contributed to the article and approved the submitted version.

## Conflict of Interest

The authors declare that the research was conducted in the absence of any commercial or financial relationships that could be construed as a potential conflict of interest.

## Publisher’s Note

All claims expressed in this article are solely those of the authors and do not necessarily represent those of their affiliated organizations, or those of the publisher, the editors and the reviewers. Any product that may be evaluated in this article, or claim that may be made by its manufacturer, is not guaranteed or endorsed by the publisher.

## Appendix A

### Interview Protocol

#### Instructions

Good morning (afternoon/evening). Thank you very much for your valuable time. We are conducting this research to examine the level of human resources professionals’ effectiveness in the Telco industry of Pakistan. The research aims to recognize the factors and elements that contribute to their effectiveness. During the interview, you will be asked to rate yourself on the basis of your effectiveness being an HR professional in the organization. There is no right or wrong or desirable or undesirable answer. We would like you to feel comfortable with saying what you really think and how you really feel about your effectiveness. Please explain thoroughly the reasons or justifications of being more effective or less effective as it will help us to achieve research objectives of the current study. If it is acceptable with you, we would like to tape record our conversation. The purpose of this is so that we can get all the valuable information but, at the same time, be able to carry on an attentive conversation with you. We assure you that this information will only be used for academic purposes. Thank you.

1. **Demographics and work information**

• How long have you been working with the current company?
• How long have you been in this field/in the current position?
• May I know your highest qualification as regards to your current position?
• Could you please explain your roles and responsibilities in your current position?

2. **Human resources professionals’ effectiveness**

• Could you please explain the contributing factors and elements that determine your effectiveness?
• Please rate below factors on the scale of 1 to 100%. And explain the reasons of your effectiveness.

**Being a human resources professional, I am effective…**

Less effective Most effective

1% ——————————————————————————> 100%

1. ….in recruiting the right candidates at the right time.

Please give your justification……………………………

1. ….in response to fulfilling the needs of an employee.

Please give your justification……………………………

1. ….in communicating timely information.

Please give your justification……………………………

1. ….in developing HR policies and procedures.

Please give your justification……………………………

1. ….in performing optimum HR practices.

Please give your justification……………………………

1. ….in their responsibilities to meet the expectations of employees.

Please give your justification……………………………

1. ….in their roles to meet the expectations of employees.

Please give your justification……………………………

1. ….in providing career plans for the employees.

Please give your justification……………………………

1. ….in providing support and services for the employees.

Please give your justification……………………………

*Thank you very much for participating in the interview process. Your time is very much appreciated, and your inputs have been very helpful.*

## References

[B1] Ahmad A. Ahmad A. (2014). A study of learning practices intelecommunication sector: evidence from Faisalabad. *Int. J. Manag. Organ. Stud.* 3 13–19. 10.1001/jamaoncol.2021.6987 34967848 PMC8719276

[B2] Ahmad A. Kausar A. R. Azhar S. M. (2015). HR professionals’ effectiveness and competencies: a perceptual study in the banking sector of Pakistan. *Int. J. Bus. Soc.* 16 201–220.

[B3] Ahmad A. Sharif M. Y. Kausar A. R. (2012). “Preliminary study of the moderating effect of organizational support on the relationship between HR competencies, HR willingness and HR professionals’ effectiveness link: a Study of Line Manager in a Pakistani Banking Sector,” in *Proceedings of the 2nd International Conference on Business Management*, Islamabad.

[B4] Ahmad A. Sharif M. Y. Kausar A. R. (2014). HR professionals’ competencies and performance in the banking sector of Pakistan. *World Appl. Sci. J.* 31 2001–2009.

[B5] Ahmad A. Sharif M. Y. Kausar M. A. R. (2013). A comparative study of the HR professionals’ effectiveness in the banking sector of Pakistan. *Res. J. Instit. Bus. Admin. Karachi Pak.* 8 98–115. 10.54784/1990-6587.1289

[B6] Armstrong M. (2011). *Armstrong’s Handbook of Strategic Human Resource Management.* London: Kogan Page Publishers.

[B7] Balagam A. Fariduddin S. (2008). A survey of major HR challenges in the mature and emerging industries a comparison between IT and banking industries of Pakistan. *J. Independ. Stud. Res. Manag. Soc. Sci. Econ.* 6 24–30. 10.31384/jisrmsse/2008.06.2.5

[B8] Barney J. B. (1991). Firm resources and sustained competitive advantage. *J. Manag.* 17 10–15.

[B9] Barney J. Wright M. Ketchen D. J. (2001). The resource-based view of the firm: ten years after 1991. *J. Manag.* 27 625–641. 10.1177/014920630102700601

[B10] Boxall P. Purcell J. (2011). *Strategy and Human Resource Management.* London: Palgrave Macmillan.

[B11] Boyce M. S. (2006). Scale for resource selection functions. *Divers. Distrib.* 12 269–276. 10.1111/j.1366-9516.2006.00243.x

[B12] Castillo-Montoya M. (2016). Preparing for interview research: the interview protocol refinement framework. *Qual. Rep.* 21 811–831.

[B13] Choi S. L. Ismail W. K. W. Amin S. M. (2010). An exploratory study on the Malaysian human resource professionals in the manufacturing sector. *Int. J. Bus. Soc.* 11 89–105.

[B14] Cohen M. Z. Kahn D. L. Steeves D. L. (2000). *Hermeneutic Phenomenological Research: A Practical Guide for Nurse Researchers.* Thousand Oaks, CA: Sage.

[B15] Creswell J. W. Poth C. N. (2017). *Qualitative Inquiry and Research Design: Choosing Among Five Approaches.* Thousand Oaks, CA: Sage publications.

[B16] Dessler G. (2010). *Human Resource Management: The Strategic Role of Human Resource Management.* Hoboken, NJ: Prentice Hall, Inc.

[B17] Etikan I. Musa S. A. Alkassim R. S. (2016). Comparison of convenience sampling and purposive sampling. *Am. J. Theor. Appl. Stat.* 5 1–4. 10.6224/JN.61.3.105 24899564

[B18] Fareed M. Ahmad A. Saoula O. Salleh S. S. M. M. Zakariya N. H. (2020). High performance work system and human resource professionals’ effectiveness: a lesson from techno-based firms of Pakistan. *Int. J. Innov. Creat. Change* 13 989–1003.

[B19] Fareed M. Noor W. S. Isa M. F. Shahzad A. Laeeq H. (2016c). The role of human capital development and high performance work system in sustaining the human resource professionals’ effectiveness: a lesson from Pakistan’s telco companies. *Int. J. Econ. Perspect.* 10 512–525.

[B20] Fareed M. Isa M. F. M. Noor W. S. W. M. (2016a). HR professionals’ effectiveness through human capital development, organizational culture and high performance work system: a proposed framework. *Int. Bus. Manag.* 10 1720–1728.

[B21] Fareed M. Noor W. S. Isa M. F. Salleh S. S. (2016b). Developing human capital for sustainable competitive advantage: the roles of organizational culture and high performance work system. *Int. J. Econ. Perspect.* 10 655–673.

[B22] Folkestad B. (2008). *Analyzing Interview Data Eurosphere Working Paper Series*, Vol. 13. Bergen: University of Bergen.

[B23] Gehman J. Glaser V. L. Eisenhardt K. M. Gioia D. Langley A. Corley K. G. (2017). Finding theory–method fit: a comparison of three qualitative approaches to theory building. *J. Manag. Inq.* 27 1–18. 10.4135/9781071878682.n4

[B24] Gentles S. J. Charles C. Ploeg J. McKibbon K. A. (2015). Sampling in qualitative research: insights from an overview of the methods literature. *Qual. Rep.* 20 1772–1789.

[B25] Guest D. Conway N. (2011). The impact of HR practices, HR effectiveness and a ‘strong HR system’on organizational outcomes: a stakeholder perspective. *Int. J. Hum. Resour. Manag.* 22 1686–1702. 10.1080/09585192.2011.565657

[B26] Guest G. Bunce A. Johnson L. (2006). How many interviews are enough? An experiment with data saturation and variability. *Field Methods* 18 59–82. 10.1177/1525822x05279903

[B27] Hafeez S. Hong L. L. Saeed B. B. Afsar B. (2017). Customer knowledge management as a success driver for business in mobile sector of Pakistan. *Int. Rev. Manag. Market.* 7 1–14.

[B28] Han J. Chou P. Chao M. Wright P. M. (2006). The HR competencies-HR effectiveness link: a study in Taiwanese high-tech companies. *Hum. Resour. Manag.* 45 391–406. 10.1002/hrm.20114

[B29] Huselid M. A. (1995). The impact of human resource management practices on turnover, productivity, and corporate financial performance. *Acad. Manag. J.* 38 635–672. 10.5465/256741

[B30] Huselid M. A. Jackson S. E. Schuler R. S. (1997). Technical and strategic human resources management effectiveness as determinants of firm performance. *Acad. Manag. J.* 40 171–188. 10.5465/257025 257025

[B31] Hussain T. Asif S. (2012). Is employees’ turnover intention driven byorganizational commitment and perceived organizational support. *J. Qual. Technol. Manag.* 8 1–10.

[B32] Imtiaz S. Y. Khan M. A. Shakir M. (2015). Telecom sector of Pakistan: potential, challenges and business opportunities. *Telemat. Inf.* 32 254–258. 10.1016/j.tele.2014.09.002

[B33] Inyang B. J. (2010). Strategic human resource management (SHRM): a paradigm shift for achieving sustained competitive advantage in organization. *Int. Bull. Bus. Admin.* 7 23–36.

[B34] Jacob S. A. Furgerson S. P. (2012). Writing interview protocols and conducting interviews: tips for students new to the field of qualitative research. *Qual. Rep.* 17 1–10.

[B35] Kabene S. M. Orchard C. Howard J. M. Soriano M. A. Leduc R. (2006). Theimportance of human resource management in health care: a global context. *Hum. Resour. Health* 4 1–17. 16872531 10.1186/1478-4491-4-20 PMC1552082

[B36] Kadir K. A. Noor W. S. W. M. (2015). Students’ awareness of the importance of english language proficiency with regard to future employment. *World Rev. Bus. Res.* 5 259–272.

[B37] Kazmi S. Kanwal F. Rathore K. Faheem K. Fatima A. (2021). The relationship between transformational leadership and organisational learning capability with the mediating role of perceived human resource effectiveness. *South Asian J. Hum. Resour. Manag.*, 8, 133–157.

[B38] Khan A. A. Abbasi S. O. B. H. Waseem R. M. Ayaz M. Ijaz M. (2016). Impact of training and development of employees on employee performance through job satisfaction: a study of telecom sector of Pakistan. *Bus. Manag. Strat*. 7, 29–46. 10.5296/BMS.V7I1.9024

[B39] Khan M. A. Rehman K. Rehman I. Safwan N. Ahmad A. (2011). Modeling Link between Internal Service Quality in Human Resource Management and Employees Retention: a case of Pakistani Privatized and Public Sector Banks. *Afr. J. Bus. Manag.* 5, 949–959.

[B40] Kim S. Ryu S. (2011). Social capital of the HR department, HR’s change agent role, and HR effectiveness: evidence from South Korean firms. *Int. J. Hum. Resour. Manag.* 22 1638–1653. 10.1080/09585192.2011.565649

[B41] Kirwan C. Birchall D. (2006). Transfer of learning from managementdevelopment programmes: testing the Holton model. *Int. J. Train. Dev.* 10 252–268. 10.1111/j.1468-2419.2006.00259.x

[B42] Malik S. Waheed A. Tufail S. Zameer H. Hussain M. (2012). Issues andproblems faced by organizations in recruitment: a case of telecom sector inPakistan. *Int. J. Bus. Manag. Tomorrow* 2 1–7.

[B43] Mansoor M. Fida S. Nasir S. Ahmad Z. (2011). The impact of job stress on employee job satisfaction a study on telecommunication sector of Pakistan. *J. Bus. Stud. Q.* 2 50–56.

[B44] McMahan G. C. Virick M. Wright P. M. (1999). Alternative theoretical perspectives for strategic human resource management revisited: progress, problems, and prospects. *Res. Person. Hum. Resour. Manag.* 4 99–122.

[B45] Ministry of Finance, Government of Pakistan (2014–2013). *Pakistan Economic Survey.* Pakistan: Government of Pakistan.

[B46] Mitsuhashi H. Park H. J. Wright P. M. Chua R. S. (2000). Line and HR executives’ perceptions of HR effectiveness in firms in the People’s Republic of China. *Int. J. Hum. Resour. Manag.* 11 197–216. 10.1080/095851900339828

[B47] Morse J. M. (2005). Evolving trends in qualitative research: advances in mixed-method design. *Qual. Health. Res.* 15 583–585. 10.1177/1049732305275169 15802536

[B48] Pope C. Van Royen P. Baker R. (2002). Qualitative methods in research on healthcare quality. *Qual. Saf. Health Care* 11 148–152. 10.1136/qhc.11.2.148 12448807 PMC1743608

[B49] Priem R. L. Butler J. E. (2001). Is the resource-based “view” a useful perspective for strategic management research? *Acad. Manag. Rev.* 26 22–40. 10.5465/amr.2001.4011928

[B50] PTA (2013–2012). *Pakistan Telecommunication Authority (Annual Report, 2013-2012).* Available online at: http://www.pta.gov.pk/annual-reports/annreport2013_1.pdf (accessed November 17, 2020).

[B51] PTA Annual Report (2016). *Pakistan Telecommunication Authority (Annual Report, 2016-2015).* Available online at: http://www.pta.gov.pk/ann_report_171116.pdf (accessed November 17, 2020).

[B52] Rana T. M. Salaria M. R. Herani G. M. Amin M. (2009). Identifying factors playing important role in the increasing employees’ turnover rate: a caseof telecom industry in Pakistan. *Ind. J. Manag. Soc. Sci.* 3 80–89.

[B53] Razzaq S. Aslam U. Bagh T. Saddique S. (2017). The impact of human resources management practices on employee commitment: evidence from Pakistan telecom sector. *Int. J. Acad. Res. Bus. Soc. Sci.* 7 649–667.

[B54] Richard O. C. Johnson N. B. (2001). Strategic human resource management effectiveness and firm performance. *Int. J. Hum. Resour. Manag.* 12 299–310. 10.1080/09585190121674

[B55] Ruel H. J. M. Bondarouk T. V. Velde M. V. (2007). The contribution of e-HRMto HRM effectiveness: results from a quantitative study in a Dutch ministry. *Emp. Relat.* 29 280–291. 10.1108/01425450710741757

[B56] Ryu S. Kim S. (2013). First-line managers’ HR involvement and HR effectiveness: the case of South Korea. *Hum. Resour. Manag.* 52 947–966. 10.1002/hrm.21576

[B57] Schuler R. (1990). Repositioning the human resource function: transformation or demise? *Acad. Manag. Perspect.* 4 49–60. 10.5465/ame.1990.4274667

[B58] Schuler R. S. Jackson S. E. (2005). Quarter-century review of human resource management in the U.S.: the growth in importance of the international perspective. *Manag. Rev.* 16 11–35. 10.5771/0935-9915-2005-1-11 20113525

[B59] Sharif M. Y. Ahmad A. Kausar A. R. (2011). A comparative study on the effectiveness of human resource professionals in Pakistan and Malaysia. *IBIMA Bus. Rev.* 1–11. 10.5171/2011.728528

[B60] Shoaib M. Noor A. Tirmizi S. R. Bashir S. (2009). “Determinants of employeeretention in telecom sector of Pakistan,” in *Proceedings of the 2nd COMSATS International BusinessResearch Conference (November 14, 2009)*, Lahore.

[B61] Snell S. A. Youndt M. A. Wright P. M. (1996). Establishing a framework for research in strategic human resource management: merging resource theory and organizational learning. *Res. Person. Hum. Resour. Manag.* 14 61–90.

[B62] Sultan N. Wajid A. Omar A. B. Waseem M. Rustam S. (2012). Human resource (HR) initiatives and their impact on performance of national bank of Pakistan. *Sci. Ser. Data Rep.* 4 44–57.

[B63] Sultana A. Irum S. Ahmed K. Mehmood N. (2012). Impact of training onemployee performance: a study of telecommunication sector in Pakistan. *Interdiscip. J. Contemp. Res. Bus.* 4:646.

[B64] Ulrich D. Allen J. Brockbank W. Younger J. Nyman M. (2009). *HR Transformation: Building Human Resources from the Outside in.* New York, NY: Sage.

[B65] Ulrich D. Lake D. (1991). Organizational capability: creating competitive advantage. *Executive* 5 77–92. 10.5465/ame.1991.4274728

[B66] Ulrich D. Brockbank W. Johnson D. Sandholtz K. Younger J. (2008). *Human Resource Competencies: Mastering at the Intersection of People and Business.* Alexandria, VA: Society for Human Resource Management.

[B68] Wright P. M. McMahan G. C. Snell S. A. Gerhart B. (2001b). Comparing line and HR executives’ perceptions of HR effectiveness: services, roles, and contributions. *Hum. Resour. Manag.* 40 111–123. 10.1002/hrm.1002

[B67] Wright P. M. Dunford B. B. Snell S. A. (2001a). Human resources and the resource based view of the firm. *J. Manag.* 27 701–721. 10.1177/014920630102700607

[B69] Wright P. M. McMahan G. C. McCormick B. Scott S. W. (1998). Strategy, core competence, and HR involvement as determinants of HR effectiveness and refinery performance. *Hum. Resour. Manag.* 37 17–29. 10.1002/(sici)1099-050x(199821)37:1<17::aid-hrm3>3.0.co;2-y

[B70] Zikmund W. (2003). *Business Research Methods*, 7th Edn. Mason, OH: Thomson/South-Western.

